# Interactive youth science workshops benefit student participants and graduate student mentors

**DOI:** 10.1371/journal.pbio.3000668

**Published:** 2020-03-30

**Authors:** Pallavi Kompella, Brant Gracia, Lucy LeBlanc, Shelly Engelman, Chinmayee Kulkarni, Niral Desai, Viviana June, Stephen March, Sarah Pattengale, Gabriel Rodriguez-Rivera, Seung Woo Ryu, Isabel Strohkendl, Pooja Mandke, Greg Clark

**Affiliations:** 1 Division of Pharmacology and Toxicology, College of Pharmacy, The University of Texas at Austin, Austin, Texas, United States of America; 2 Department of Genetics, The University of Texas MD Anderson Cancer Center, Houston, Texas, United States of America; 3 Department of Molecular Biosciences, College of Natural Sciences, The University of Texas at Austin, Austin, Texas, United States of America; 4 Texas Institute for Discovery Education in Science, College of Natural Sciences, The University of Texas at Austin, Austin, Texas, United States of America; 5 Department of Physics, College of Natural Sciences, The University of Texas at Austin, Austin, Texas, United States of America; 6 Department of Electrical and Computer Engineering, The University of Texas at Austin, Austin, Texas, United States of America; 7 Department of Chemical Engineering, The University of Texas at Austin, Austin, Texas, United States of America

## Abstract

Science communication and outreach are essential for training the next generation of scientists and raising public awareness for science. Providing effective science, technology, engineering, and mathematics (STEM) educational outreach to students in classrooms is challenging because of the need to form partnerships with teachers, the time commitment required for the presenting scientist, and the limited class time allotted for presentations. In our Present Your Ph.D. Thesis to a 12-Year Old outreach project, our novel solution to this problem is hosting a youth science workshop (YSW) on our university campus. The YSW is an interpersonal science communication and outreach experience in which graduate students from diverse scientific disciplines introduce middle and high school students to their cutting-edge research and mentor them to develop a white-board presentation to communicate the research to the workshop audience. Our assessment of the YSW indicated that participating young students expressed significantly more positive attitudes toward science and increased motivation to work in a STEM career after attending the workshop. Qualitative follow-up interviews with participating graduate students’ show that even with minimal time commitment, an impactful science communication training experience can be achieved. The YSW is a low-cost, high-reward educational outreach event amenable to all disciplines of science. It enhances interest and support of basic science research while providing opportunities for graduate students to engage with the public, improve their science communication skills, and enhance public understanding of science. This YSW model can be easily implemented at other higher education institutions to globally enhance science outreach initiatives.

## Abstract 2

This Community Page article describes Youth Science Workshops (YSWs) - a novel science education outreach event in which graduate students from different disciplines communicate their research to young students. Assessment of the outcomes suggest that this workshop benefits all the participants, increasing science interest for students and improving the science communication skills of the graduate student presenters.

## Introduction

Science, technology, engineering, and mathematics (STEM) careers are notable in that they tend to have higher than average growth, wages, and educational requirements than other career paths. Additionally, almost all STEM occupations were estimated to grow rapidly from 2014 to 2024, with mathematical science occupations expected to grow by 28% compared with the average growth for all other occupations of 6.5% [1]. Standardized testing of STEM-interested students suggests a deficiency in science education. For example, in 2017, of the high school students who took the standardized American College Testing (ACT) exam, used for college admission in the United States, 68% failed to meet the ACT STEM benchmark despite nearly half (48%) of them having an interest in pursuing a STEM-related career. Further, this number increased to over 90% when only considering students from disadvantaged backgrounds (e.g., ethnic minorities, low income, and first generation in college) [2]. Clearly, the rapid growth of STEM fields necessitates additional educational opportunities to enhance STEM preparedness.

Science outreach events, including active and hands-on learning approaches, can help to fill this gap by increasing interest in STEM and teaching relevant scientific concepts by involving professionals who work in STEM fields [3,4,5,6]. Moreover, such events constitute an opportunity for scientists to develop science communication skills, such as the ability to break down complex concepts and deliver a concise, factual message in an engaging manner [7,8]. STEM graduate students provide a unique resource as communicators of science during outreach, because they are often at the forefront of scientific discovery and also benefit greatly from developing communication skills at this early stage of their career [9,10,11].

The Present Your Ph.D. Thesis to a 12-Year Old (abbreviated Present Your Ph.D. herein) outreach project provides graduate students with opportunities to communicate their research to nonexpert audiences and thereby improve young students’ understanding of basic science research [9]. In 2017, we hosted middle and high school students from the local Austin community at the University of Texas at Austin (UT Austin) campus to determine whether an interactive science education, youth science workshop (YSW), could enhance their attitudes and interest in science.

### YSW

In the pilot workshop, 26 middle school students and 6 graduate student mentors participated. A basic interview style survey revealed positive impact of the YSW on both the young students and their mentors, but we did not statistically assess the outcomes of the event. In 2018, we expanded the workshop, and 49 students between 4th and 10th grade from various backgrounds participated in the YSW (S1 Methods). Approximately 30% of the students were home-schooled, and the remainder of the students were from 18 different schools in and around the Austin area. Among the 49 student participants, 27% were females, 59% were males, and 14% did not wish to reveal their gender (S1 Table).

**Fig 1:**
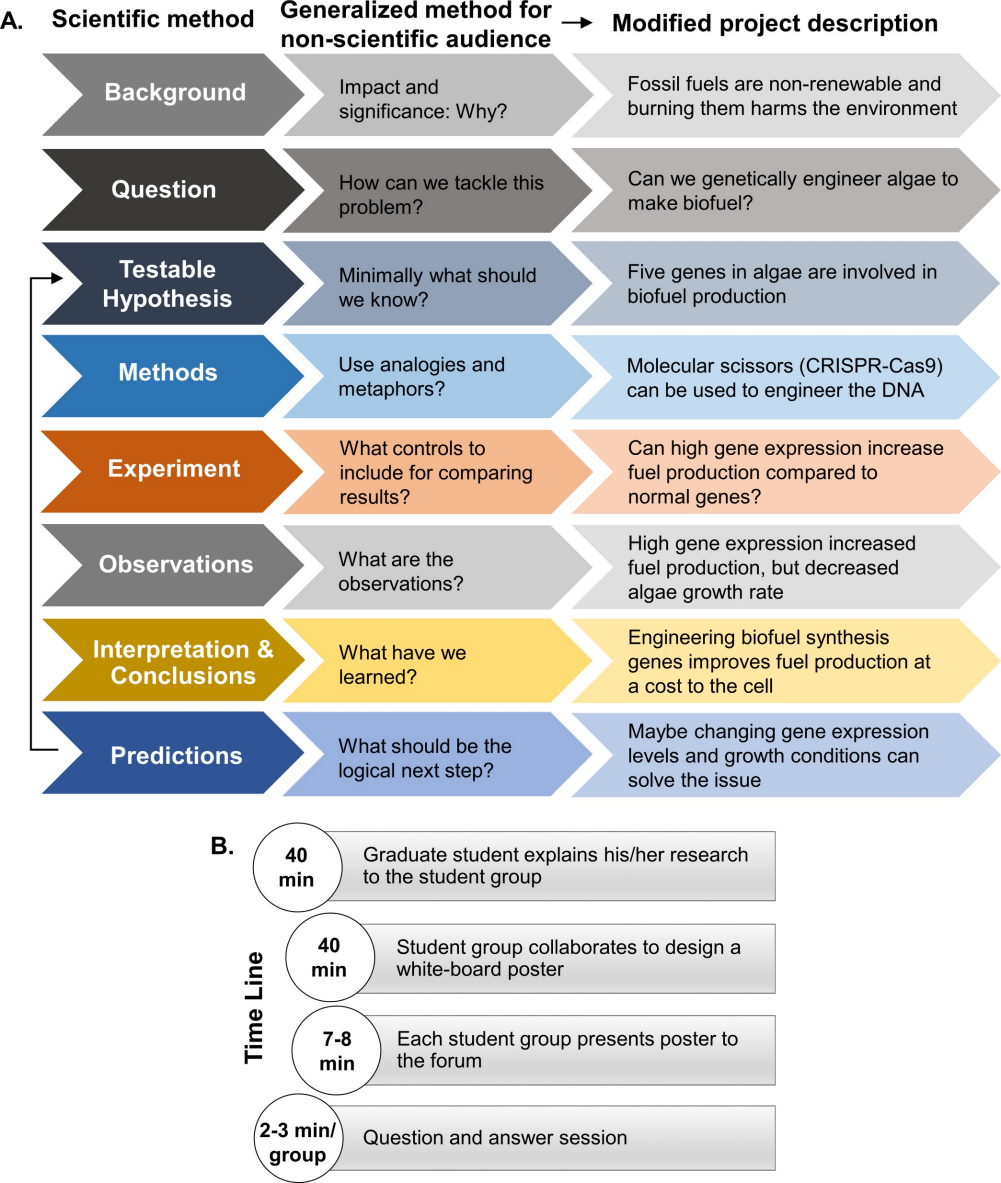
Schematic of the YSW. (A) A schematic to modify doctoral research projects for presentation to a nonscientific audience. (B) YSW time line. YSW, youth science workshop.

A few weeks prior to the event, we invited Present Your Ph.D. outreach members to participate as YSW mentors, and nine graduate students from various backgrounds of natural sciences (e.g., biology, physics) and engineering (chemical and computer) with or without prior science communication experience participated. Seven out of nine graduate students had some previous experience in education outreach through visiting local area classrooms with the Present Your Ph.D. outreach project. With the purpose of guiding graduate student presenters to effectively communicate their research to the young students in this new workshop model, we designed a research project translation guide (Fig 1A).

On the day of the event, five to six school students were grouped with each graduate student and given access to a white board and dry-erase markers. Graduate student mentors first explained their Ph.D. research work through an interactive chalk-talk type presentation on a white board, which was later erased. Then, each group of school students held a brainstorming session among themselves in order to come up with creative analogies and metaphors to explain the research topic at hand effectively. After a short snack break, the youth groups with guidance from their respective graduate student mentors, created a “white board poster” and explained the research topic to the entire forum (fellow school students, graduate students, and parents). An example of a “white board poster” created by students at the YSW is shown in S1 Fig. Each presentation was followed by a question and answer session, which was mostly answered by the young students with occasional help from their respective graduate student mentor. This format allowed a substantial opportunity for interaction and exchange of ideas between the graduate students and the participating students during the chalk-talk and science poster presentations (Fig 1B).

Assessment of the potential outcomes of science outreach activities is important in order to ensure successful science communication and gain feedback that can be used for long-term improvement of these skills [12,13,14]. To evaluate the outcomes of the YSW on students’ attitudes toward science, scientists, and academic research, we designed a pre-post retrospective survey addressing five specific educational constructs adapted from a similar study performed to assess attitudes toward mathematics [12]. The constructs included interest in science, intentions to persist in science, confidence in learning science, perceptions of scientists, and perceived usefulness of science. In addition, participating graduate students were also interviewed to assess the perceived benefits of the YSW and their overall satisfaction with the program. This study was carried out in accordance with the IRB guidelines (IRB proposal number 2018-10-0141). The investigators obtained a voluntary written parental consent and child’s assent form to participate in the study. Graduate student participants were all members of the Present Your Ph.D. outreach program and volunteered to participate in the YSW.

In addition to the positive outcomes reported in the student survey and the graduate student focus group interview, YSW appeared to have other positive impacts on its participants: (1) provided an enriched learning environment and helped build a strong science-inquiry based relationship with the local community, (2) inspired creativity in young students and encouraged them to think and ask questions, (3) helped graduate students learn the importance of science outreach and provided them with a unique opportunity to communicate their research to a lay audience. The documented outcomes as well as the suggested impacts of this short-duration outreach event indicate that the YSW is an effective new model for public engagement by science graduate students.

### YSW improves middle and high school students’ interest and attitude toward science

For the “Interest in science” construct, we found that the YSW statistically significantly increased students’ interest in science. For example, before participating in the workshop, only 53% of students said that they “strongly agree” that science is interesting compared with 76% after the YSW (Table 1 and S2A Fig). Likewise, students felt more emboldened to engage in science communication after the workshop; specifically, the YSW increased students’ interest in presenting science and hearing science presentations by 25% and 17%, respectively (Table 1, S2A and S3A Figs). Before the YSW, 24% of participating students disagreed when asked “I like asking questions,” but after the YSW, only 12% of students disagreed. This decrease in disagreement was accompanied by an increase in strong agreement of 12% after the YSW (S3A Fig). For “intention to persist in science,” 57% of students strongly agreed that they “want to take more classes in science” after the YSW compared with only 35% before the workshop (Table 1 and S2B Fig). Further, when asked about pursuing a career as a “research scientist,” 15% of the responses switched from disagree to agree or strongly agree after the YSW (Table 1 and S2B Fig). Additionally, a significant increase in agreement in response to the question “I can teach science to others” from 47% to 64% showed students’ gained confidence in understanding science and teaching others (Table 1 and S2C Fig). Finally, students expressed more positive attitudes towards scientists in general. For example, after the workshop, there was a 19% increase in students responding strongly agree to “scientists are cool” (Table 1 and S2D Fig). This result is consistent with prior reported literature that meeting scientists face to face, even for short times, can help positively influence stereotyped perceptions of scientists [8]. Overall, the survey responses demonstrate that YSW improves students’ attitude toward learning and persisting in science. Three of the seven questions that were not significant still had small positive effects using a metric for the magnitude of a treatment effect, approximately 0.2 Cohen’s effect size [15] and *p* < 0.084 (Table 1).

**Table 1:** Quantitative results of the pre-post retrospective survey for student participants in YSW. Only students who responded to the construct questions were included in the analysis. *p*-value was calculated using a two-tailed paired samples *t* test. Effect size measures for two independent groups, i.e., Cohen’s effect size: 0.2 (small); 0.5 (medium), 0.8 (large).

Construct	Survey item	pre/ post	*n*	mean	*p*-value	Strongly disagree	Disagree	Neutral	Agree	Strongly agree
						1	2	3	4	5
**Interest in science**	Science is interesting.	Pre	49	4.27	0.00361*	2%	6%	8%	31%	53%
		Post	49	4.59		2%	2%	6%	14%	76%
	I like hearing science presentations.	Pre	49	4.00	0.14180	4%	10%	24%	37%	24%
		Post	49	3.94		6%	6%	16%	31%	41%
	I like presenting science to others.	Pre	49	3.57	0.00001*	6%	12%	22%	37%	22%
		Post	49	4.04		6%	4%	16%	27%	47%
	I like asking questions.	Pre	49	3.55	0.00114*	4%	20%	20%	27%	29%
		Post	49	3.86		6%	6%	24%	22%	41%
**Intention to persist in science**	I want to take more classes in science.	Pre	49	3.73	0.00016*	4%	10%	29%	22%	35%
		Post	49	4.18		6%	2%	16%	18%	57%
	I want to be a research scientist when I grow up.	Pre	49	3.24	0.00019*	4%	31%	24%	18%	22%
		Post	49	3.61		4%	16%	24%	24%	31%
	I want to have a job in STEM.	Pre	49	4.18	0.05853	2%	6%	14%	27%	51%
		Post	49	4.35		2%	6%	14%	10%	67%
	I want to have a job not related to STEM	Pre	47	2.45	0.15952	32%	28%	17%	11%	13%
		Post	47	2.32		43%	15%	21%	11%	11%
**Confidence in learning science**	I can teach science to others.	Pre	47	3.30	0.00010*	11%	13%	30%	30%	17%
		Post	47	3.74		9%	6%	21%	30%	34%
	I understand science.	Pre	49	4.08	0.08320	2%	6%	12%	41%	39%
		Post	49	4.27		2%	4%	14%	24%	55%
	I feel I will get a good grade in science.	Pre	48	4.21	0.07325	2%	4%	13%	33%	48%
		Post	48	4.38		2%	2%	13%	23%	60%
**Perception of scientists**	Scientists are cool.	Pre	48	4.13	0.01789*	2%	8%	13%	29%	48%
		Post	48	4.40		2%	4%	13%	15%	67%
	Anyone can be a scientist.	Pre	47	3.81	0.29373	4%	6%	34%	15%	40%
		Post	47	3.94		6%	2%	30%	15%	47%
**Perceived usefulness of science**	Science is useful to the world.	Pre	46	4.63	0.37149	0%	4%	2%	20%	74%
		Post	46	4.70		2%	2%	4%	7%	85%
	Science will affect me throughout my life.	Pre	48	4.25	0.03988*	0%	6%	17%	23%	54%
		Post	48	4.46		2%	0%	13%	21%	65%

*p < 0.05.

**Abbreviations:** STEM, science, technology, engineering, and mathematics; YSW, youth science workshop

Further, we designed one of the questions to test the validity of the students’ responses by asking an inverse question from within the survey. For example, “I would like to have a job in STEM” versus “I would like to not have a job in STEM”. On posing this control question, as expected, the responses dramatically shifted to the opposite direction, demonstrating the validity of the students’ responses (Table 1 and S3B Fig). The results for the questions (“Anyone can be a scientist” and “Science is useful to the world”) were not significant and reflected small changes in attitudes toward science post YSW, suggesting that participating students might have had preconceived positive attitudes for science in these areas before YSW (Table 1, S3D and S3E Fig).

Moreover, we observed a strong interest in science among many participating students before the event (“Science is interesting”, pre YSW average = 4.27 on a 5-point Likert scale, Table 1). This suggests that the impact of the YSW could be enhanced for a student group that is less interested in science before the YSW. Doerschuk and colleagues [16] has shown that students from low socioeconomic status (SES) are less likely to succeed in STEM disciplines than students who come from more privileged backgrounds. Therefore, for future YSW events, we would like to compare the impact of the YSW on students from low SES backgrounds with STEM-focused students from higher SES backgrounds.

### YSW improves the effectiveness of science communication

To gain general insights into the effectiveness of the workshop and to make future improvements, we also polled for qualitative feedback by asking three short response questions: (a) How did YSW influence your academic/career plans? (b) What were the best part(s) of this workshop?, and (c) How can we improve YSW?, as well as a general overall rating for the event on a scale of 1 to 5 (1 = very poor to 5 = excellent). A total of 51% of students rated the YSW “excellent,” and an additional 37% rated the workshop as “good.”

When the students were asked “How did the workshop influence your academic/career plans?,” 39% of students reported a significant impact, 26% reported some impact, and 22% reported no impact (S4 and S5 Figs). Students from each group reported: “It has made me more interested in science and has given me a look at what scientists do” (significant impact), “Not a lot but still fun” (some impact), and “I learned cool stuff, but it didn’t really” (no impact). Because many students were already inclined towards STEM-based academic and career plans, 17% reported that the workshop confirmed and/or encouraged their future plans. They explained “it encouraged [them] to continue on a science-based path for [their] future career”.

When asked “the best part(s) of this workshop was…,” the students reported Learning the Content, Teamwork & Interaction, and Presenting to be the best parts (S4 and S5 Figs). The 50% of students who reported Learning the Content as the best part recounted that they enjoyed “learning and understanding the topic,” “learning what research students are doing,” and “learning several aspects of different disciplines of science.” About 28% of students who reported Teamwork & Interaction as being one of the favorite aspects of the YSW said “[one of the best parts of the workshop was] getting to work as a group and interacting with each other,” “having fun with my teacher and peers,” and “learning [from] and talking to the graduate [student].” Presenting the Ph.D. research was also reported as a popular aspect by 26% of students. They elaborated that their favorite part was “learning things [they] didn’t know and sharing [their] knowledge,” “learning and presenting the PhD,” and “the presentations because [they] got some public speaking experience and it gave [them] a chance to speak about something [they] love”.

Lastly, when asked “This workshop could be improved by…,” suggestions were collected across four categories: Logistics, Other/Non-Academic, Workshop Length, and Content-Based (S4 and S5 Figs). Although there were suggestions for improvements in all four categories, we thought the most interesting recommendation from the participants was increasing the length of the entire workshop and providing more time to prepare student-led presentations. This feedback suggests that a YSW on a larger scale, comparable to a youth science conference with more opportunities to present and interact with scientists, would be well received by local students. Further, a youth science conference would provide more graduate students the opportunity for public engagement by involving more students and parents.

### YSW helps graduate student mentors to improve their science communication skills

We also interviewed the graduate students who participated in the YSW by holding a focus group with six participants (S1 Extended Document). The students were asked questions regarding their degree of involvement with outreach in the past, the perceived benefits of the YSW, and their overall satisfaction with the program. Most of the graduate students had previously participated in the Present Your Ph.D. outreach organization for the past two years but almost exclusively in the “scientist in the classroom” model of outreach, in which interaction and overall impacts are more limited. The graduate students reported that explaining their research projects to participating students of the YSW strengthened their ability to understand the complex concepts of their science as well as break down and transmit those concepts to others verbally. Additionally, the feedback from students regarding how relevant and influential their research was for the general public was beneficial for their morale and determination to continue their work. The graduate students also indicated that their presentation and communication skills improved significantly after participating in the Present Your Ph.D. outreach program. The “Dunning-Kruger effect” is a cognitive bias in which novices or less experienced individuals wrongly overestimate their ability in a specific area, thus it is important to consider the possibility for this bias that would predict potential flaws in scientists' self-reported gains in skills like science communication [17]. However, it is worth noting that the graduate students participated in the focus group several months after the workshop event, and these graduate students did not start as “unskilled” science communicators, and both of these two factors have been suggested to minimize the bias predicted by the "Dunning-Kruger effect” [18]. One of the graduate students said that “[the event] made me appreciate how to simplify what we were working on [to] make it more digestible…good experience distilling ideas and dissertation.” All of the students that participated in the focus group were satisfied with the Present Your Ph.D. outreach program and would recommend it to a colleague.

## Discussion

There is generally a lack of formal training for graduate students in science communication, with only a few universities offering communication training courses, and quantitative evaluation of these courses is rare [19,20,21]. A recent study of outreach activity by astronomers indicated that institutional support, including training, is an important factor in determining participation in outreach [22].

Moreover, short-duration science outreach often follows the “scientist in the classroom” model, and evaluation of the outcomes of this informal type of outreach has rarely been done and is difficult to assess [10]. The short-duration outreach events are believed to have positive long-term impacts based on the idea that meeting scientist role models, learning about science, and having positive science-related experiences will affect students’ persistence in science and possibly influence their choice of science as a career [23,24]. However, thus far, there is a lack of evidence that supports these suggested positive outcomes of short-duration outreach events [25].

Here, we present and evaluate a low-cost outreach activity, YSW, in which a graduate student mentor presents their research to a group of younger students and then trains them to communicate key concepts to a wider lay audience. For 9 out of 15 questions from the pre-post retrospective survey, we found that the students that participated in the YSW showed pre-post gains in their attitudes toward science, expanded their interest beyond the scope of the event, and their perception of scientists (*p* < 0.05, S2 Fig and Table 1). A majority of the participating students reported that their favorite aspects of the workshop were learning the science content, interacting in small teams, and having an opportunity to present to their peers. The positive impact on the students suggests that the graduate students effectively communicated their complex research ideas to a young nonscientific audience and stimulated general interest in the science enterprise.

The graduate students expressed that this experience solidified their interest in academia and also proposed that future events should target schools with fewer funds and resources to enhance the effectiveness of the YSW. For most graduate students, the use and design of the pre-post retrospective survey of the participants and the inclusion of a post-workshop focus group aligns with recent research resulting in guidelines for evaluating and improving the efficacy of science communication [26].

## Conclusions

In summary, YSW is an example of a low-cost and low-time investment outreach experience that can be easily replicated at other institutions. For example, Rice University adapted this very model to conduct its first YSW in August 2019. We found that this type of event, apart from substantially improving students’ attitudes toward science and scientists, can also improve the public’s perception of science and increase their trust in STEM professionals. Furthermore, the YSW gave both younger students and graduate students a valuable opportunity to practice science communication skills, which are immensely helpful for boosting both their interest and preparedness in STEM careers and engagement with the public.

## Supporting information

S1 Methods (PDF)

S1 Table Demographics of student participants. A total of 49 students from different ethnic backgrounds studying in 4th through 10th grades, either homeschooled or from 20 different schools in and around Austin area participated in YSW. DWA, do not wish to answer, YSW, youth science workshop.
(TIF)

S1 Fig A representative picture of a “white board poster” created by student participants’ at the YSW. YSW, youth science workshop.
(TIF)

S2 Fig YSW improves the middle and high school students’ interest and attitude toward science. A representative survey question from each evaluated construct is shown with the corresponding data (A–E). Each bar graph shows the change in the percentage of responses pre and post workshop. Inset graphs show change in percentage of responses for each question (red, Strongly Disagree; orange, Disagree; gray, Neutral; light green, Agree; dark green, Strongly Agree). *p*-value was calculated using a paired samples *t* test, and *p* < 0.05 was considered statistically significant. YSW, youth science workshop.
(TIF)

S3 Fig Quantitative results for other questions of the pre-post retrospective survey for student participants in YSW. A representative survey question from each evaluated construct is shown with the corresponding data (A–E). Each bar graph shows the change in the percentage of responses pre and post workshop. Inset graphs show change in percentage of responses for each question (red, Strongly Disagree; orange, Disagree; gray, Neutral; light green, Agree; dark green, Strongly Agree). *p*-value was calculated using a paired samples *t* test, and *p* < 0.05 was considered statistically significant. YSW, youth science workshop.
(TIF)

S4 Fig YSW improves the effectiveness of science communication. Students’ open-ended responses to the following questions: (A) How did YSW influence your academic/career plans? (B) What were the best part(s) of this workshop? (C) How can we improve YSW? The responses were categorized using thematic coding to generate labels for assigning units of meaning to descriptive information. YSW, youth science workshop.
(TIF)

S5 Fig Data reduction for qualitative survey responses. (TIF)

S1 Extended Document Graduate student survey. (PDF)

## Abbreviations

SES: socioeconomic status
STEM: science, technology, engineering, and mathematics
YSW: youth science workshop
