# Supplementary material for: Interactive youth science workshops benefit student participants and graduate student mentors
Source: PLoS Biol. 2020 Mar 30;18(3):e3000668. doi: 10.1371/journal.pbio.3000668 (PMC7145268; doi:10.1371/journal.pbio.3000668)
Supplement: S1 Methods — (PDF) [file pbio.3000668.s001.pdf]

Methods

Participant recruitment

We used a flyer to invite 10 to 16-year-old middle and high school students for the YSW. This flyer included the event details, schedule of the workshop, and an online registration link for students and parents. The flyer was posted on social media and also emailed randomly to various local schools in and around Austin. We also leveraged the established network of the Present Your Ph.D. outreach and Shadow a Scientist programs (9).

All the school students in the solicited age-group irrespective of their age, gender, ethnicity/race, which school they study in and socioeconomic status were included in the study.

Data Collection

Data collected from participants without the parental consent/child assent form was not included in the study. No participant identifiable data was collected and participants were given the opportunity to exit the study without any penalty. Participants were not required to answer questions, when appropriate items included the option "do not wish to answer (DWA)". Participants were asked if they would like to include their responses in the study after completing the survey. If they responded no, their responses were not included.

Six graduate student mentors were interviewed to assess the impacts of their participation in the YSW. One student mentor was not available to be interviewed due to a scheduling conflict. In order to exclude confirmatory bias, qualitative data collected from two graduate student mentors who also happened to be the investigators of the study was not included.

Measures

To assess student response change from before the workshop (pre) to after the workshop (post), a standardized retrospective pre/post survey in printed form was

employed (12). The survey was disseminated to school students during the last 5-10 minutes of the workshop and were given 15 minutes to write down their responses.

Data Analysis

De-identified survey data was processed using both Excel and SPSS. To assess attitudinal change from pre to post, a two-tailed paired samples t-test was employed. Effect size measures for two independent groups (Cohen’s Effect Size) were calculated as described. Likewise, descriptive statistics of students' responses on all survey items was computed.

The responses to the qualitative short response survey questions were analyzed using thematic coding procedures described previously (13). Specifically, students’ qualitative data were analyzed using three procedures: data reduction, data display, and conclusion drawing/ verification to derive the major codes or conceptual categories to group the students’ fractional responses.

Students’ qualitative responses sometimes aligned with more than one thematic category. As a result, the percentages may not add up to 100%. Only students who responded to the open-ended survey questions were included in the analysis.
