# Supplementary figures and images for: Interactive youth science workshops benefit student participants and graduate student mentors
Source: PLoS Biol. 2020 Mar 30;18(3):e3000668. doi: 10.1371/journal.pbio.3000668 (PMC7145268; doi:10.1371/journal.pbio.3000668)

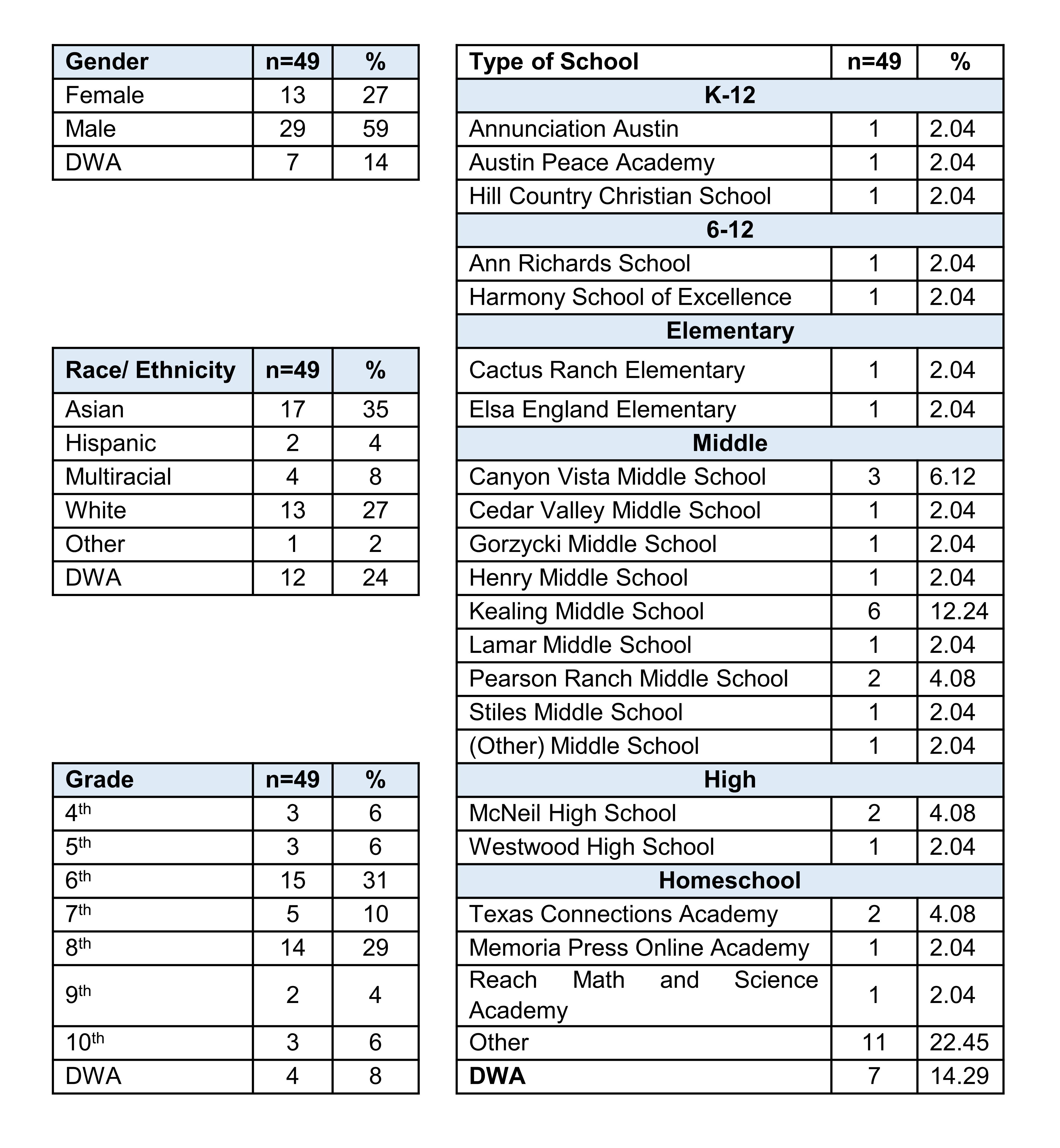

Supplement: S1 Table — A total of 49 students from different ethnic backgrounds studying in 4th through 10th grades, either homeschooled or from 20 different schools in and around Austin area participated in YSW. DWA, do not wish to answer, YSW, youth science workshop. (TIF) [file pbio.3000668.s002.tif]

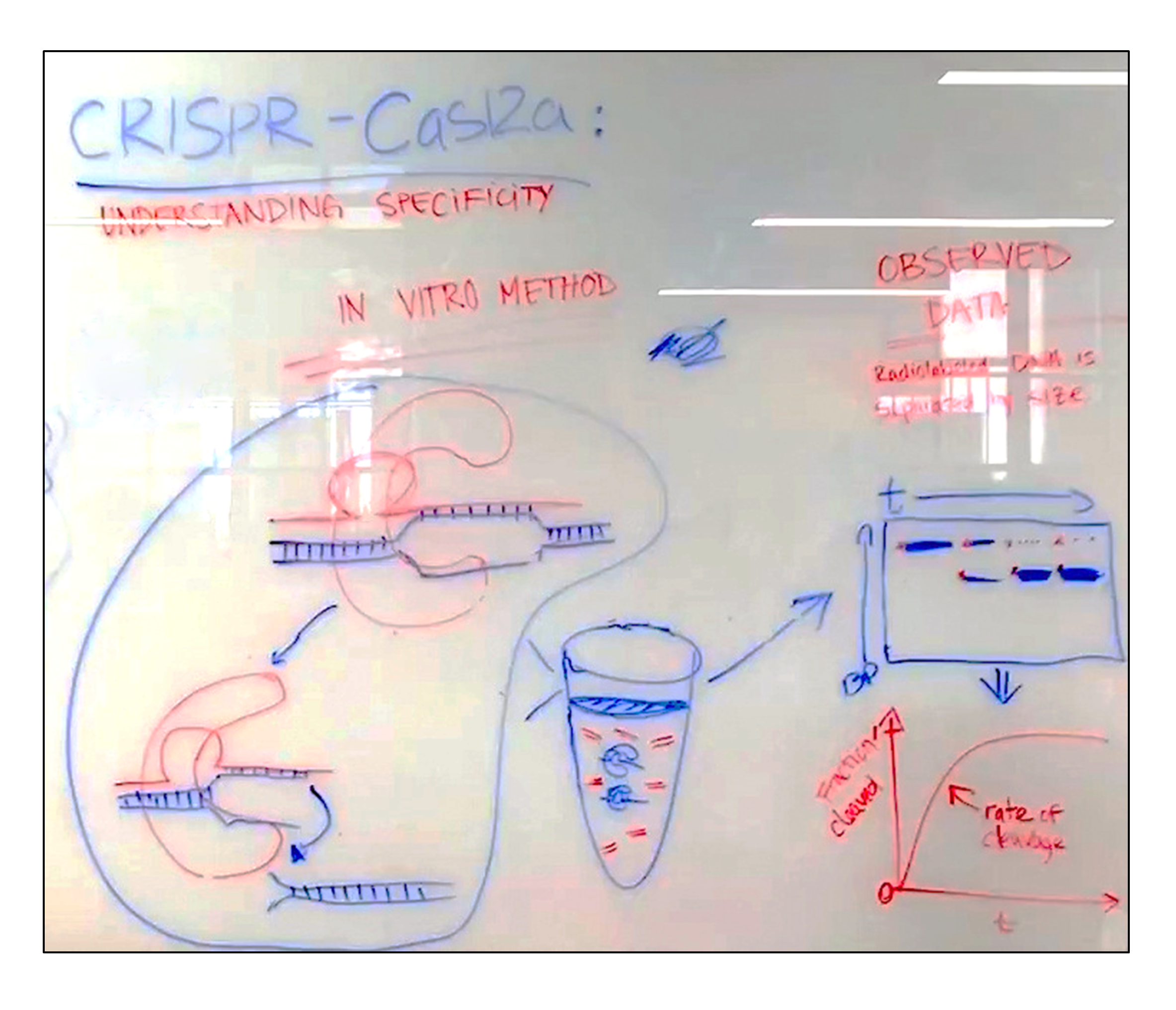

Supplement: S1 Fig — YSW, youth science workshop. (TIF) [file pbio.3000668.s003.tif]

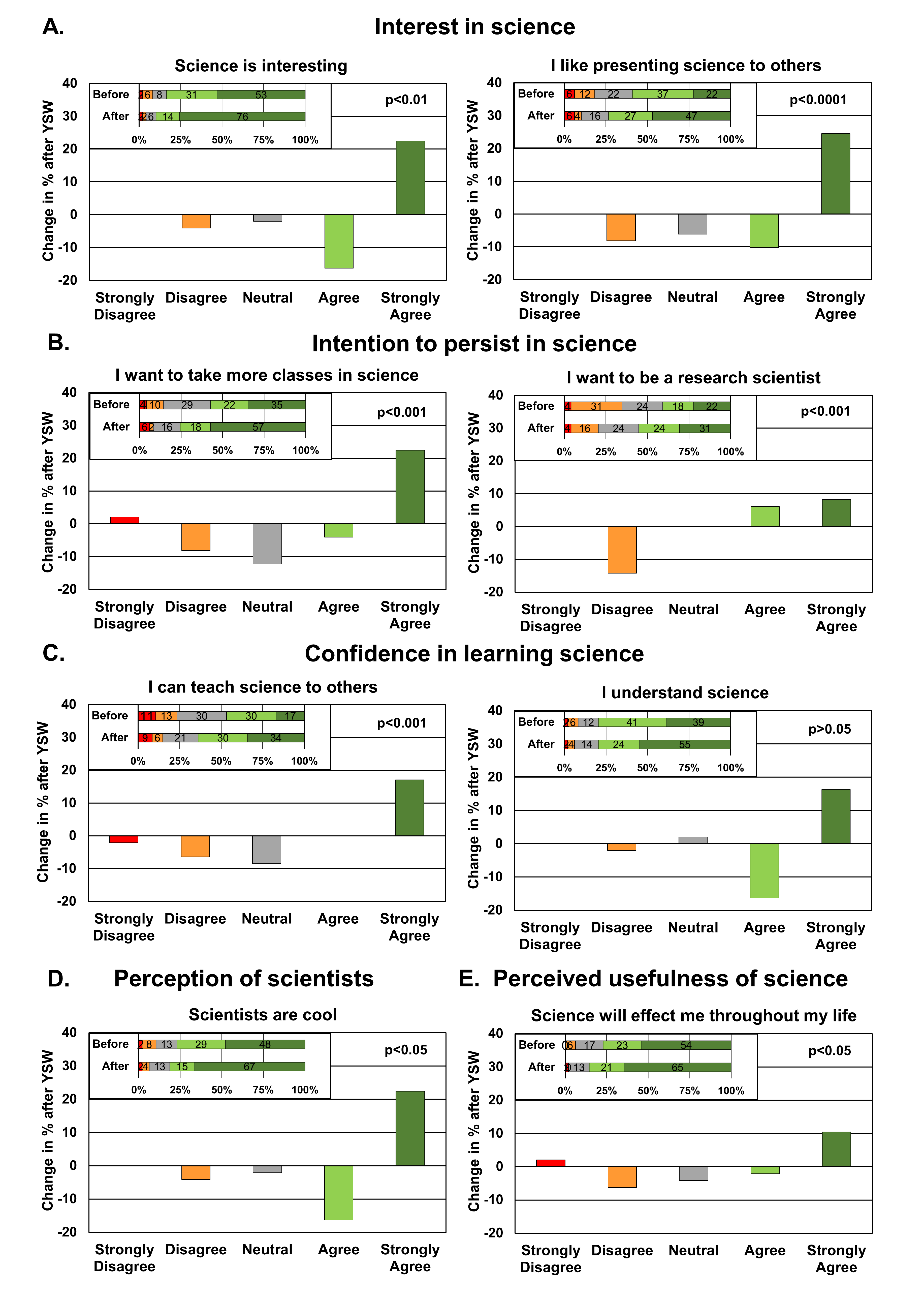

Supplement: S2 Fig — A representative survey question from each evaluated construct is shown with the corresponding data (A–E). Each bar graph shows the change in the percentage of responses pre and post workshop. Inset graphs show change in percentage of responses for each question (red, Strongly Disagree; orange, Disagree; gray, Neutral; light green, Agree; dark green, Strongly Agree). p-value was calculated using a paired samples t test, and p < 0.05 was considered statistically significant. YSW, youth science workshop. (TIF) [file pbio.3000668.s004.tif]

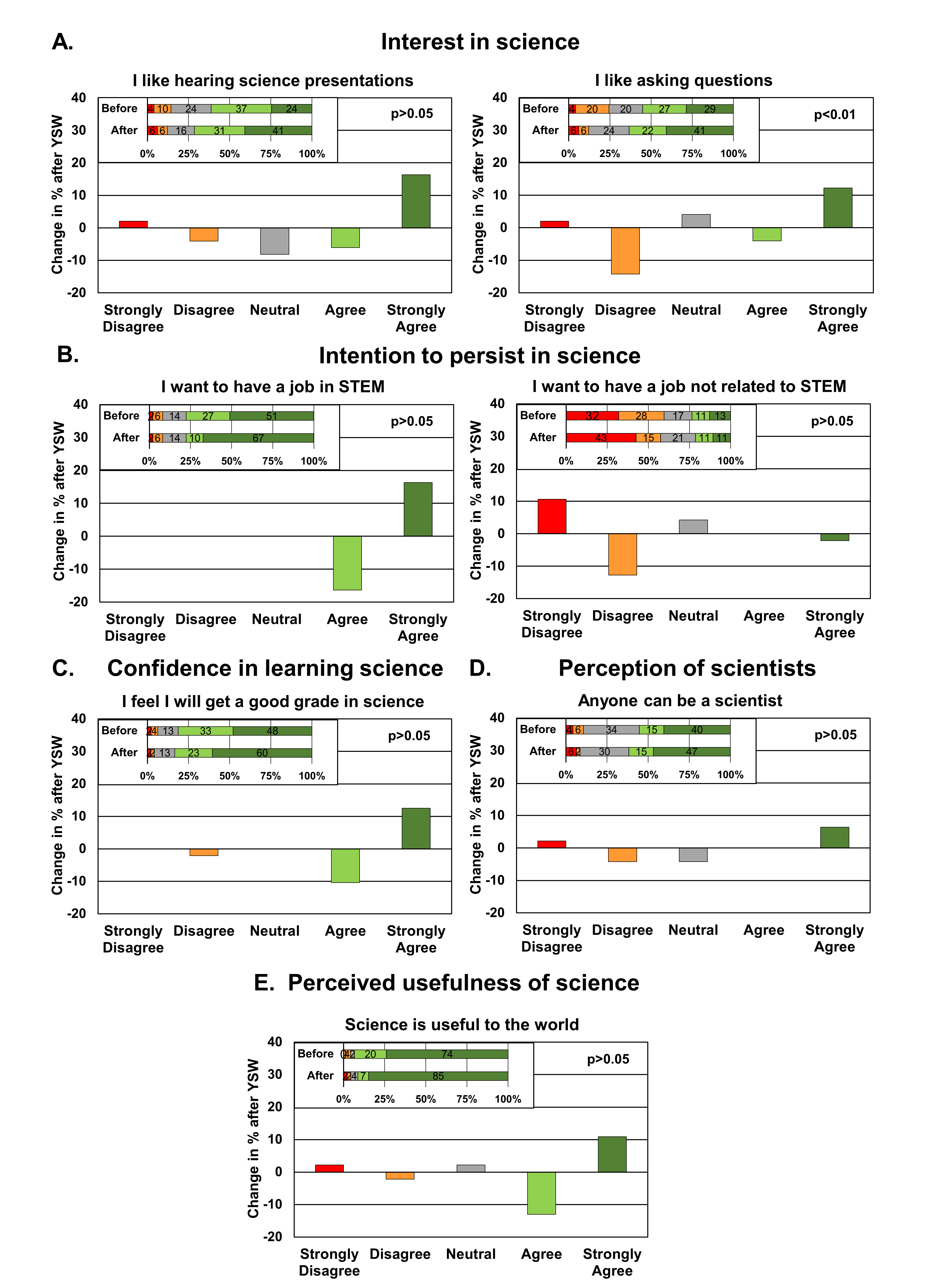

Supplement: S3 Fig — A representative survey question from each evaluated construct is shown with the corresponding data (A–E). Each bar graph shows the change in the percentage of responses pre and post workshop. Inset graphs show change in percentage of responses for each question (red, Strongly Disagree; orange, Disagree; gray, Neutral; light green, Agree; dark green, Strongly Agree). p-value was calculated using a paired samples t test, and p < 0.05 was considered statistically significant. YSW, youth science workshop. (TIF) [file pbio.3000668.s005.tif]

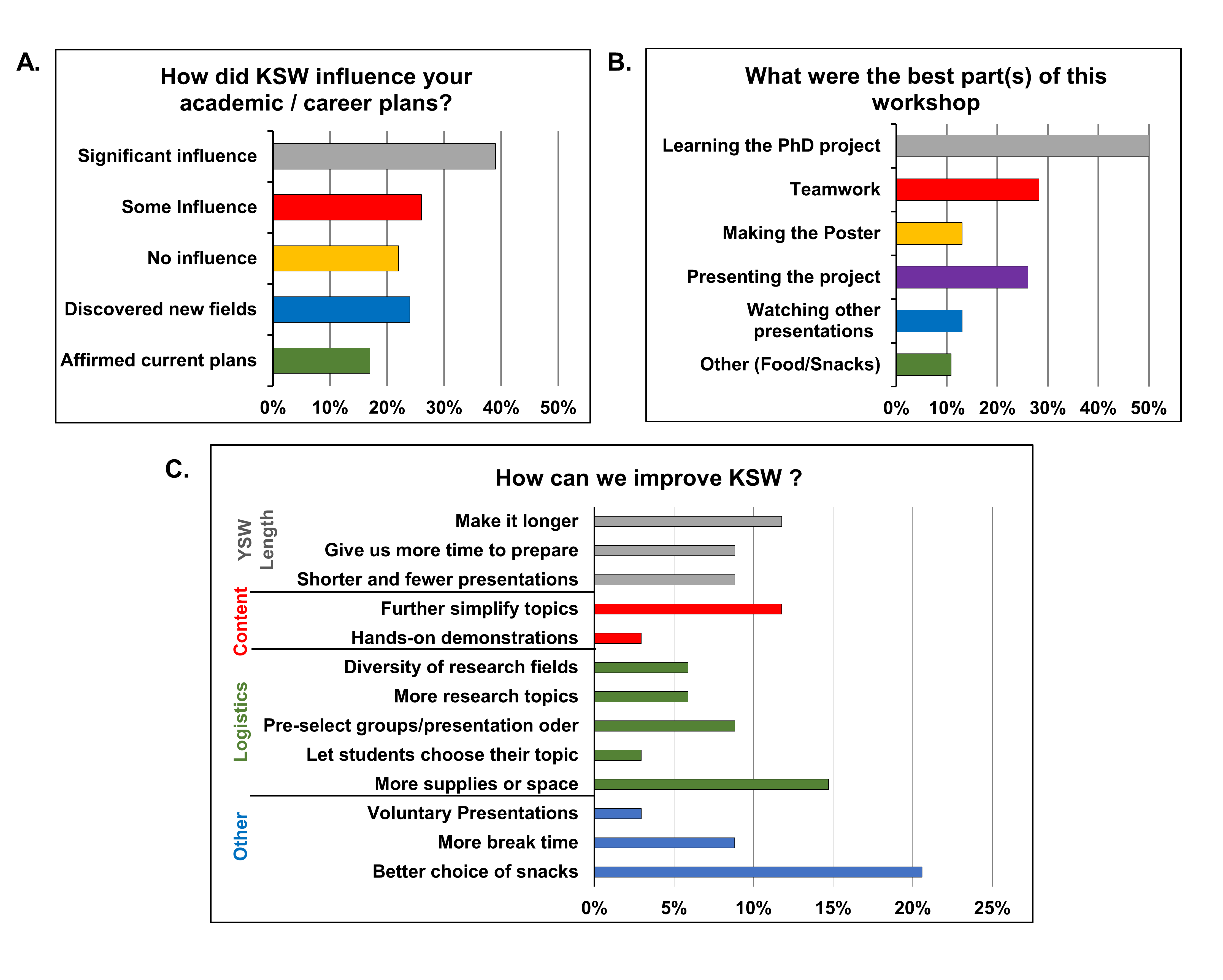

Supplement: S4 Fig — Students’ open-ended responses to the following questions: (A) How did YSW influence your academic/career plans? (B) What were the best part(s) of this workshop? (C) How can we improve YSW? The responses were categorized using thematic coding to generate labels for assigning units of meaning to descriptive information. YSW, youth science workshop. (TIF) [file pbio.3000668.s006.tif]

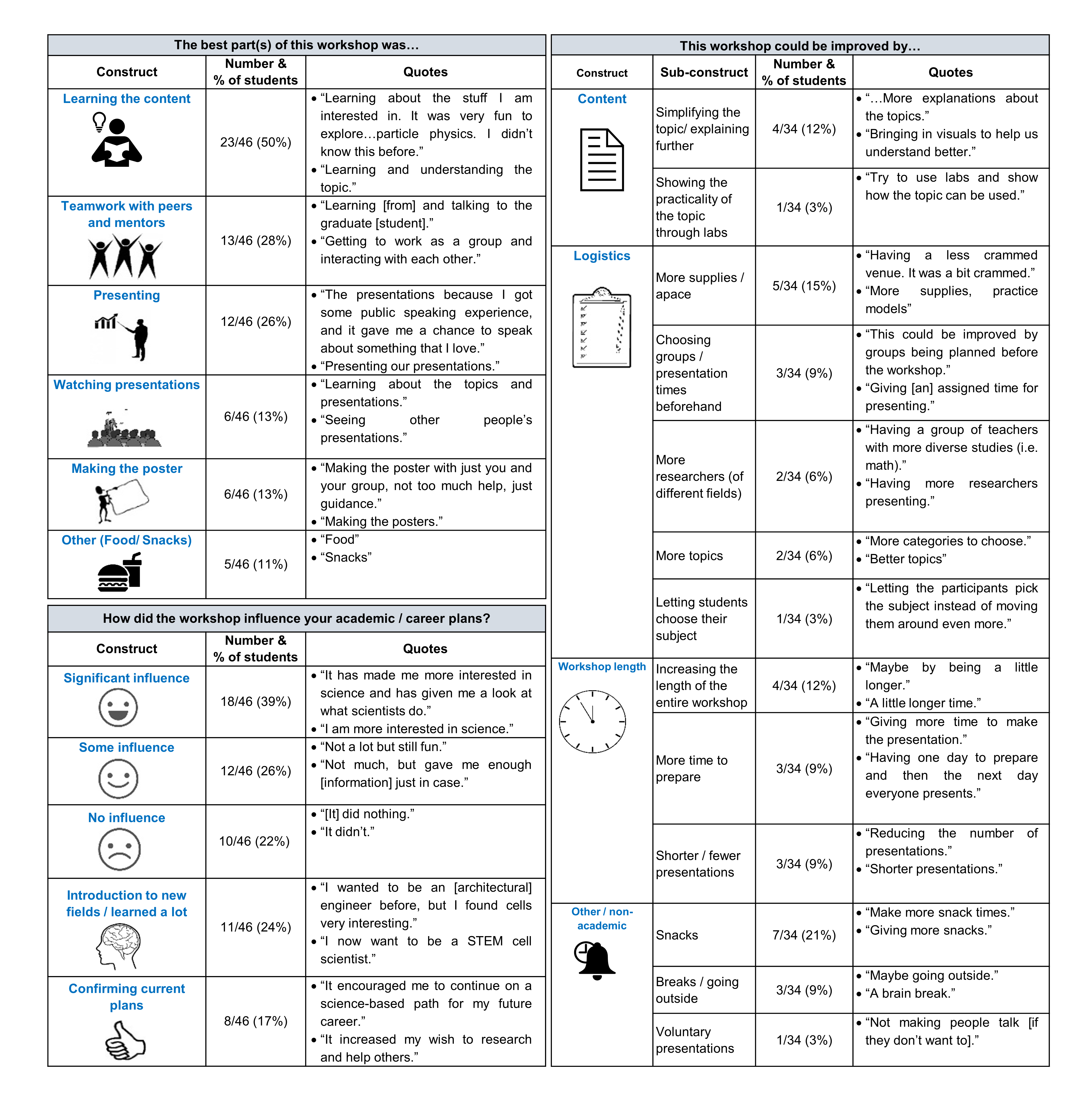

Supplement: S5 Fig — (TIF) [file pbio.3000668.s007.tif]
