# Supplementary material for: Interactive youth science workshops benefit student participants and graduate student mentors
Source: PLoS Biol. 2020 Mar 30;18(3):e3000668. doi: 10.1371/journal.pbio.3000668 (PMC7145268; doi:10.1371/journal.pbio.3000668)
Supplement: S1 Extended Document — (PDF) [file pbio.3000668.s008.pdf]

Present Your PhD: Focus Group Notes (March 4, 2019)

The Present Your PhD Focus Group was held on March 4, 2019 at 5pm in BIO 214, and six graduate students participated. The students were asked questions regarding their degree of involvement, perceived benefits of the program, challenges they encountered, their overall satisfaction with the program, and the support they received. The students also provided suggestions for improvement which are also included below.

Degree of Involvement

Most of the graduate students have participated in the Present Your PhD workshops for the past two years.

- **Can you tell me a little bit about your involvement in the "Present your PhD" workshops?**
    - Not very involved, had a talk at Kealing Middle School
    - Participated 3-4 times at Kealing Middle School
    - Has been participating for about 2 years
    - Has been participating for about 2 years
    - Has participated twice
    - Quotes:
        - "I haven't been very involved, but I have been aware of it for a while."
        - "Started with PYP program around May or June last year and went to Kealing Middle School about three or four times."

Benefits

The graduate students explained that they were able to breakdown and better understand their research projects after having to explain it to the elementary and middle school children. They found it beneficial to have received honest feedback about how interesting and influential their research was for the general public. The students also felt that their presentation and communication skills improved significantly after participating in the PYP program. For most students, this experience solidified their interest in academia.

- **What did you get out of the experience?**
    - **How did this experience impact the way you see your research project?**
        - Made the [research project] more digestible,
        - "If I can help [the kids] understand, I can help anyone [understand]"
        - Liked getting feedback from what the kids took away because kids are honest about what they learned and what they didn't
        - Liked hearing about what the general public found interesting about their research
        - Helped the graduate students think about delivering the message correctly and find out what science is important
        - Quotes:
            - "Made me appreciate how to simplify what we were working on and make it more digestible … explaining to a 10-12-year-old much more difficult than explaining to an adult so it makes you go back to the basics…good experience distilling ideas and dissertation."
            - "If you want a picture of what the general public thinks of your research, talking to kids in particular is one of the best things because they will be

        honest and they are also relatively uninformed because they haven't been exposed to all that much."

- **To what extent did this experience improve your communication skills, if at all?**
    - had to go back to basics (from technical jargon to everyday words), improved presentation skills
    - Helped one of the graduate students perform well on a qualitative exam
    - Quotes:
        - "It has definitely improved my presentation skills a lot…gaining experience of explaining technical jargon [using] everyday words gave me more confidence about what I was talking about."
- **How has this experience impacted your career goals, if at all?**
    - No more interest in academic research, now one of the graduate students wants to pursue being a K-12 teacher ("total career change")
    - Solidified interest in public outreach and the research they are involved in
    - Increasing desire to teach not maybe full-time but part-time
    - Quotes:
        - "It changed my [career goals]. I have no interest in academic research anymore. I have decided that I have more of an interest in K12 teaching…It was [therefore] a really useful experience in that respect."
        - "More of [continuing along the same path] …was able to get involved with public outreach."
        - "Didn't change my interest…but solidified it."
        - "I have enjoyed working with the kids and explaining to them my field…it's cool to see the kids walk away and think there's more to the world than I what I thought."

Challenges

The graduate students mostly voiced concerns about logistical issues regarding the PYP workshops. They would have preferred an event with more structure in terms of assigning groups and more time to prepare the kids for the presentations.

- **What were some challenges that you encountered?**
    - Timing was a challenge (15-20 minutes)
    - Figuring out what is the most useful thing that could be explained to the students
    - Difficult to create a storyline for the kids
    - Too much time given to for the graduate students' presentation and not enough time to prepare the student's presentations
    - Difficult to get all of the kids to present or write on the board
    - Difficult to distill information especially things like math
    - Logistical challenge of kids switching groups in the middle
    - Didn't have good representation from all departments specifically physics
    - Difficult to understand whether or not the graduate students should focus on answering the kid's questions or not answer them as well due to the scope of the presentation (the graduate students did not want to confuse the kids while they were preparing their PhD presentations)
    - Need more supplies (markers) to make better models and diagrams
    - Want to get to know the students but there isn't enough time to do so

- Quotes:
  - "From all the stuff that we are doing, what is the most useful thing that we can present to [the kids] so that they can get the message and understand"
  - "I think creating a storyline because if your presentation consists of significance, introduction, techniques, and results…how are they going to follow along with what you say"

Satisfaction

All of the students that participated in the focus group were satisfied with the PYP program and would recommend it to a colleague.

- **Given your experience, would you recommend this to a fellow graduate student?**
  - All said yes
- **What would you tell them are the benefits of involvement and the challenges?**
  - They feel good after the kids get excited
  - Like seeing the transfer of information
- **What are some tips that you would give them to prepare for this?**
  - Some people feel like it is too big to explain so they use more props and diagrams
  - Props to get creative
  - Have main bullet points (significance, importance, conclusions) at hand
  - Minimize jargon and keep it simple
  - Boil down to the essence of what it is, (ex. diagonalizing a matrix to a rubix cube) analogies are great
  - Get a gauge for what is appropriate for their age range
  - Have it be more open-ended (ex. Gravity-based topic can start off like What do you know about gravity?), important to encourage dialogue
  - Make sure all of the kids are heard and feel open to asking questions
  - Explain the information to family members that are not familiar with the topic, practice in front of general people
  - Find time for a break when explaining to the kids and ask how they are doing
  - Quotes:
    - "I have [recommended this program] many times"
    - "I recommend it 1) because you feel good after seeing kids get excited about whatever you're talking about or seeing the transfer of information and 2) you feel like oh okay I just accomplished this great!"

Support

The students felt that they had not received much support in regards to preparation for the workshops, but they learned a lot from their practices with other PYP presenters and the feedback from the schools they visited.

- **What kind of support did you receive in preparing for the workshops?**
  - Not much help at all
  - Dr. Clark's recorded presentation was helpful
  - Practicing with the other PYP students
  - Getting feedback from the schools themselves was good support
  - Experience being a TA helped a lot

- Quotes:
    - “None specifically for this”
    - “Before I presented in front of a classroom, I did a trial presentation in front of other PYP members and they did give me some good feedback”

Suggestions for Improvement

The graduate students’ main suggestions for improvement included ways to improve the preparation and delivery of the PYP presentations.

- Would be helpful to have a practice talk night with the other graduate student presenters
- More supplies
- Better organization (assigning students to graduate students beforehand and not making so many switches)
- Offer tag along opportunities so the graduate students can learn from their peers
- Make a promotional video for the graduate students about what the program is all about, how to prepare, and a snip of an actual presentation (this will encourage more graduate students to participate and be more effective)
- Give the graduate students an outline of what to say, how to present (would address many questions)
- Carve out time for a panel Q&A, allow the students to ask anything about science, what they like, have guided questions to get it going
- Have the graduate students take a picture of their workspace showing them what they wear in the lab, pictures of what they do, this will help kids visualize what the graduate students do on a daily basis
- Bring up things that are real careers like research
- Some graduate students suggested including childhood pics and telling the kids stories about how they got where they are now
- Target schools with low funds or ones that don’t have as many resources because the current set of schools have a different perception of who a scientist is and what they do
- Some students suggested allowing the children to have an opportunity to get their energy out (go and walk)
